# Nondestructive estimation of leaf chlorophyll content in banana based on unmanned aerial vehicle hyperspectral images using image feature combination methods

**DOI:** 10.3389/fpls.2025.1536177

**Published:** 2025-02-26

**Authors:** Weiping Kong, Lingling Ma, Huichun Ye, Jingjing Wang, Chaojia Nie, Binbin Chen, Xianfeng Zhou, Wenjiang Huang, Zikun Fan

**Affiliations:** ^1^ National Engineering Research Center for Satellite Remote Sensing Applications, Aerospace Information Research Institute, Chinese Academy of Sciences, Beijing, China; ^2^ Key Laboratory of Earth Observation of Hainan Province, Hainan Research Institute, Aerospace Information Research Institute, Chinese Academy of Sciences, Sanya, China; ^3^ Key Laboratory of Digital Earth Science, Aerospace Information Research Institute, Chinese Academy of Sciences, Beijing, China; ^4^ School of Forestry, Hainan University, Haikou, China; ^5^ College of Life Information Science and Instrument Engineering, Hangzhou Dianzi University, Hangzhou, China; ^6^ State Key Laboratory of Remote Sensing Science, Aerospace Information Research Institute, Chinese Academy of Sciences, Beijing, China

**Keywords:** leaf chlorophyll content, banana, image feature combinations, machine learning, unmanned aerial vehicle hyperspectral imagery

## Abstract

**Introduction:**

Nondestructive quantification of leaf chlorophyll content (LCC) of banana and its spatial distribution across growth stages from remotely sensed data provide an effective avenue to diagnose nutritional deficiency and guide management practices. Unmanned aerial vehicle (UAV) hyperspectral imagery can document abundant texture features (TFs) and spectral information in a field experiment due to the high spatial and spectral resolutions. However, the benefits of using the fine spatial resolution accessible from UAV data for estimating LCC for banana have not been adequately quantified.

**Methods:**

In this study, two types of image features including vegetation indices (VIs) and TFs extracted from the first-three-principal-component-analyzed images (TFs-PC1, TFs-PC2, and TFs-PC3) were employed. We proposed two methods of image feature combination for banana LCC inversion, which are a two-pair feature combination and a multivariable feature combination based on four machine learning algorithms (MLRAs).

**Results:**

The results indicated that compared to conventionally used VIs alone, the banana LCC estimations with both proposed VI and TF combination methods were all significantly improved. Comprehensive analyses of the linear relationships between all constructed two-pair feature combinations and LCC indicated that the ratio of mean to modified red-edge sample ratio index (MEA/MSR_re_) stood out (*R* ^2^ = 0.745, RMSE = 2.17). For multivariable feature combinations, four MLRAs using original or two selected VIs and TFs-PC1 combination groups resulted in better LCC estimation than the other input variables. We concluded that the nonlinear Gaussian process regression model with the VIs and TFs-PC1 combination selected by maximal information coefficient as input achieved the highest accuracy in LCC prediction for banana, with the highest *R* ^2^ of 0.776 and lowest RMSE of 2.04. This study highlights the potential of the proposed image feature combination method for deriving high-resolution maps of banana LCC fundamental for precise nutritional diagnosing and operational agriculture management.

## 1 Introduction

Banana is one of the important tropical fruits in China; it has been widely cultivated in many regions of China, such as Hainan Province, where banana planting has become one of the pillar industries and a major source of incomes for local farmers. In recent years, the banana cultivation industry shows a relatively stable increasing trend, with a planting area of 3.3 × 10^4^ ha and an annual production of fresh fruits of 1.125 × 10^6^ t in 2023. With the increasing competition in the fruit market, the consumer demand for fruit quantity and quality is also improving, which undoubtedly puts forward higher requirements for the local fruit industry in their production management capacity. However, for decades, the inappropriate application of chemical fertilizer in pursuit of high yields has resulted in serious soil and groundwater pollution, soil quality degradation, nutrient imbalance in the banana plants, and a decline in fruit quality (Guo et al., 2010; Huang et al., 2022). Therefore, to benefit the government and land managers in making informed decisions on agricultural practices, it is critical to improve the monitoring ability and accurately diagnose the nutritional status of banana plants.

The phenotype of banana leaves is broad and big; they are the most direct organ for identifying nutrient deficiencies and guiding fertilizer application because they are the main site of photosynthesis, which determines the primary processes occurring within the plant. Leaf chlorophyll, as the key photosynthetic pigment, can absorb light energy and transfer it into the photosynthetic apparatus, providing essential energy for the growth and development of plants (Curran et al., 1992). In addition, nitrogen is an important component of chlorophyll, so monitoring leaf chlorophyll content (LCC) can indirectly indicate nitrogen and nutritional status (Haboudane et al., 2008). LCC can also be related to plant stress and senescence since it tends to decrease when a plant suffers from external stress (e.g., fertilizer shortage or pest and disease) (Chappelle et al., 1992). Quantifying LCC, overall, has aroused great attention from both land managers and ecophysiologists.

Compared to traditional chemical analysis measured in the laboratory, remote sensing technology has been proven to be an effective way to assess vegetation LCC due to its advantages of rapid data acquisition and nondestructive and accurate monitoring at a large scale (Sims and Gamon, 2002; Wu et al., 2008). Optical sensors embedded on satellites and airborne platforms can acquire spectral information over large areas, which have long been used for vegetation monitoring. Although an increasing number of optical satellite images are freely available, the use of satellite imagery for LCC quantification is still limited by the fact that the image quality is usually susceptible to atmospheric conditions (e.g., clouds and suspended particles), and the spatial resolutions and revisit frequencies are seemingly not enough for supporting crop management activities at the field level and over short critical growth stages (Inoue et al., 2016; Jay et al., 2019; Pimmasarn et al., 2020). During the recent decades, unmanned aerial vehicle (UAV) equipped with RGB, multispectral and/or hyperspectral sensor has been attracting more and more attention particularly in agriculture. RGB and multispectral data are widely used by researchers to estimate LCC for various crops (Caruso et al., 2017; Xu et al., 2022; Barata et al., 2023). However, the broadband spectral information provided by RGB and the multispectral sensors that offer average spectral information over a wide range may result in loss of critical and subtle spectral features which are available in specific hyperspectral bands (Tao et al., 2020). Unlike RGB and multispectral observations, hyperspectral data containing full- and narrow-band spectral radiation information could describe various characteristics associated with the biochemical and physiological traits of targets (Sims and Gamon, 2002; Kong et al., 2022). Consequently, a hyperspectral sensor mounted on UAV will hold a promising potential in the accurate assessment of LCC. Earlier studies have made lots of attempts to extract the spectral response characteristic of LCC at visible light and near-infrared and analyze their ability in characterizing the growth and nutritional state of crops (Haboudane et al., 2002; Inoue et al., 2016). They established LCC estimation models and achieved acceptable accuracy and performance under specific conditions for wheat, maize, peanut, and other field crops (Qi et al., 2021; Zhang et al., 2021; Qiao et al., 2022). It has been accepted that UAV mounted with hyperspectral sensor is very flexible to adjust its flight heights and efficient to collect data at a higher spatial resolution (can reach up to the centimeter level) in a short time, enabling it to capture images of various vegetations with abundant spectral and textural information during each key growth period. These properties make it more suitable for monitoring vegetation nutritional status especially in cloudy and rainy regions, such as Hainan Province. In comparison to the abovementioned field crops with relatively homogeneous canopies, fruit trees have more complex canopy structure and leaf morphology. At present, studies on the nutritional diagnosis of fruit trees from remote sensing data mainly focused on apple, pear, citrus, etc (Osco et al., 2019; Azadnia et al., 2023; Huang et al., 2024). However, for a tropical fruit tree such as banana, adoption of UAV hyperspectral remote sensing in LCC estimation remains largely unexplored.

Vegetation index (VI), calculated by the spectral reflectance of two or more bands according to linear or nonlinear mathematical formula, is widely applied for leaf biochemical parameters estimation owing to its simplicity and computational efficiency. A number of VIs were proposed based on knowledge of the reflectance properties of LCC and have been proven to have the ability of reducing the noise in hyperspectral reflectance caused by soil background, atmospheric absorption, and other leaf components, consequently maximizing the corresponding information on leaf variables of target (Rondeaux et al., 1996; Sims and Gamon, 2002; Wu et al., 2008). Lots of studies have been devoted to developing the linear relationship between VI and LCC (Gitelson et al., 2006; Wu et al., 2008; Kong et al., 2017a, Kong et al., 2017b). However, some studies showed that nonlinear models have more obvious advantages than linear models in quantitative prediction (Kira et al., 2015; Zhang et al., 2021). Lately, machine learning regression algorithms (MLRAs), e.g., Gaussian process regression (GPR), support vector regression (SVR), and adaptive regression splines (ARS), become powerful candidates for the estimation of LCC from spectral reflectance of multi-related bands or VIs because of their ability to perform adaptive, nonlinear data fitting (Verrelst et al., 2013a; Upreti et al., 2019; Guo et al., 2022). However, the VIs utilize only a limited amount of information available in spectral data and are susceptible to saturation at high canopy coverage (Kong et al., 2017a). Texture features (TFs) describe the grayscale properties and spatial arrangement of image pixels, can make up for the insensitivity of spectral information in the regional size and direction, and have a strong resistance to image noise (Khosravi and Alavipanah, 2019). To maximize the advantages of VIs and TFs, previous studies tried to combine both image features to track the variations in nutritional parameters. Some researchers extracted the VIs and TFs from remote sensing images and developed new combined image features, which were proven to provide better result for estimating LCC and nitrogen (Chen et al., 2019; Zheng et al., 2020; Guo et al., 2022). Inspired by the approved LCC modeling presented above, MLRAs seem also to be a useful tool for coupling the spectral and textural information to monitor crops (Khosravi and Alavipanah, 2019; Li et al., 2023; Biswal et al., 2024; Zhang et al., 2024). The high spatial resolution of UAV makes it possible to document abundant texture feature information in field experiments. However, the benefits of using the fine spatial resolution accessible from UAV imagery as well as the potential ability of the combined use of VIs and TFs for retrieving the LCC in banana plants, and what degree the image feature combinations can contribute to improve banana LCC compared to individual spectral features which are rarely reported and kept unknown and need to be investigated.

The aim of this study was to propose the approaches of spectral and texture feature combination based on UAV hyperspectral data then benefit the nondestructive estimation of banana LCC. The specific objectives were to (i) investigate the correlation between banana LCC and individual VI and TF and identify the optimal image features, (ii) develop two-pair feature combinations of VI and TF and establish the linear relationship with LCC, (iii) estimate the LCC using multiple MLRAs with the VIs, original VIs and TFs combination as well as selected VIs and TFs combination as input, and (iv) evaluate the potential ability of the best linear two-pair VI and TF combination and nonlinear MLRA models in banana LCC prediction and map its spatial distribution using UAV hyperspectral images.

## 2 Materials and methods

### 2.1 Study site

**Figure 1:**
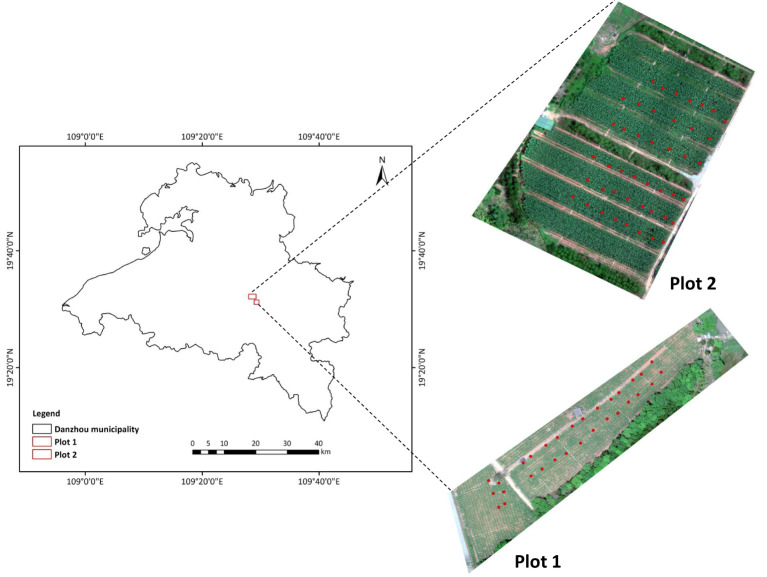
Location of study site. The red spots are the ground measurement locations for leaf chlorophyll content.

The experiment was conducted at the Banana Cultivation Research and Development Base in Danzhou Municipality (19°23′ N, 109°58′ E), Hainan Province, during April 2024. A cultivar of banana (*Musa acuminate*, AA) was selected, and two plots were investigated in this study, referred to as plot 1 and plot 2, respectively (**Figure 1**). Because the banana plants of the two plots were planted at different times, they have different growth stages during the field campaign, i.e., leaf development stage (BBCH 18) and fruit development stage (BBCH 72). The banana plants in plot 1 had eight completely open leaves, with a homogenous yellowish green color, and the blades were relatively small and narrow, while the plants in plot 2 had 11 or 12 leaves, and the fruits were already formed, the leaves were all healthy and dark green, with the area approximately twice bigger than those at the leaf development stage. The soil is laterite, with nutrient content of about 10.78 g/kg of organic matter, 60.1 mg/kg available nitrogen, 25.41 mg/kg of available phosphorus, and 120.79 mg/kg of available potassium and pH of 5.28. All plots were managed in the same way, including the fertilization and irrigation treatments.

### 2.2 Data collection

#### 2.2.1 UAV hyperspectral image acquisition and processing

The DJ M300RTK UAV platform equipped with the X20P hyperspectral imaging sensor (Cubert GmbH, Ulm, Baden-Württemberg, Germany) was used to acquire remote sensing data of the banana fields. The quality of the X20P hyperspectral imaging sensor is 630 g, and the size is 6 cm*10.7 cm*9.5 cm. Its spectral resolution is approximately 3.96 nm, with spinning wavelength of 350 to 1,000 nm with 164 bands. Radiometric calibration was taken before each flight. The flight height was approximately 80 m, and the spatial resolution of collected hyperspectral images was 2.87 cm. Its flying speed was 6 m/s, the forward overlap was about 90%, and the lateral overlap was about 90%. The flight was conducted under clear sky conditions between 11:00 a.m. and 13:00 p.m. (Beijing local time) to minimize shadowing in the images. The Cubert Utils Touch software was used for image radiometric calibration and fusion of the hyperspectral data and the corresponding panchromatic image. Agisoft PhotoScan (Agisoft, St. Petersburg, Russia) was employed to image mosaicking.

#### 2.2.2 Leaf chlorophyll content measurement

**Figure 2:**
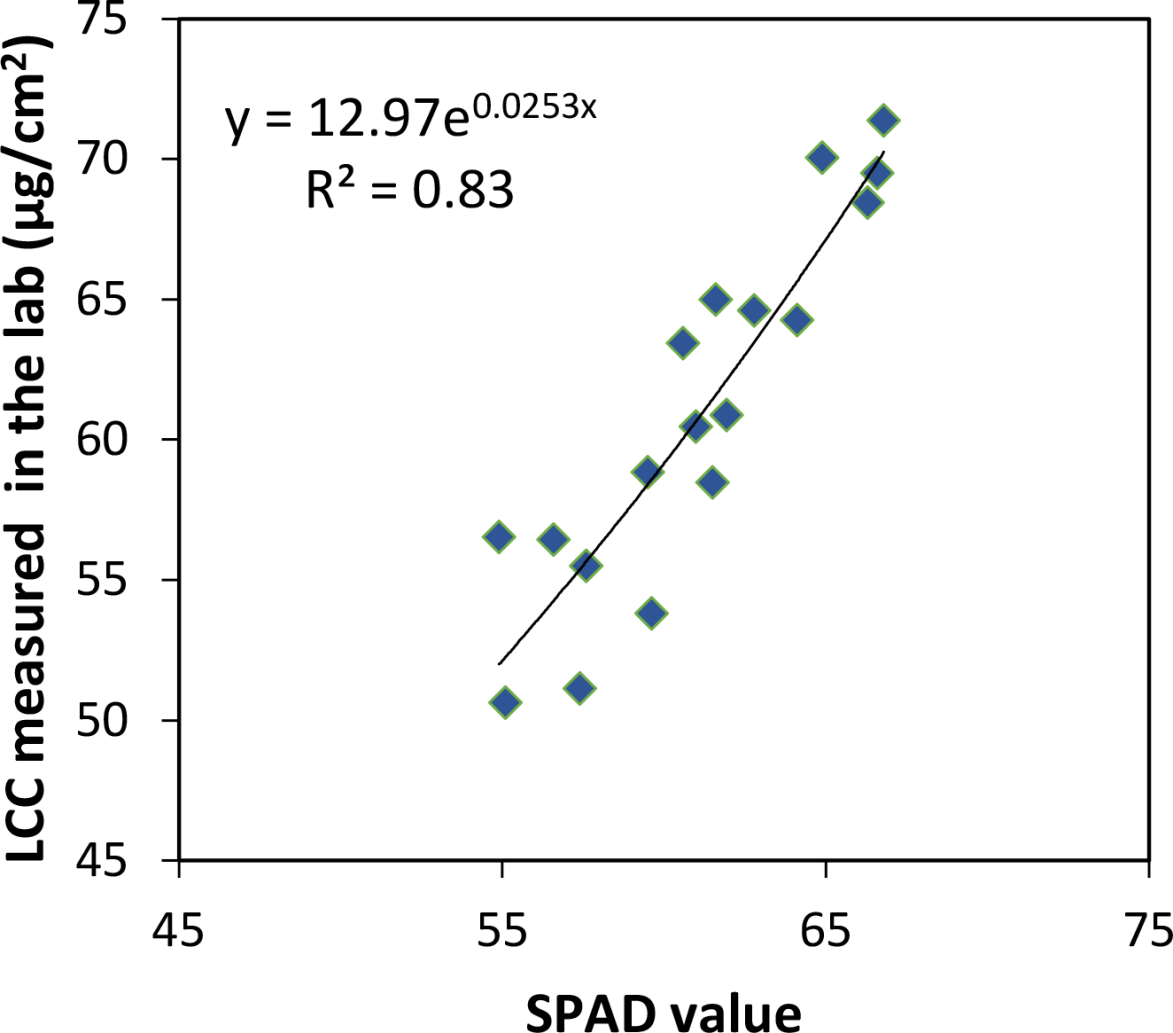
Verification of SPAD values using leaf chlorophyll content measured by chemical analysis in the laboratory.

The SPAD-502 meter (Konica-Minolta, Tokyo, Japan) measures the transmission of red (approximately 650 nm) and near-infrared (approximately 940 nm) radiation through plant leaves (Minolta, 1989). The increase of LCC could increase the absorption of red radiation, and the transmission of near-infrared radiation is used as a reference, so the calculated SPAD value was applied to represent the amount of LCC present in the sample leaf in many studies (Parry et al., 2014; Zhou et al., 2020; Zhang et al., 2022). In this study, LCC of banana was measured by the SPAD-502 meter. The central position of each measurement point was geo-located with GPS, and a total of 74 ground measurement points (30 points in plot 1 and 44 points in plot 2) were marked in **Figure 1**. The SPAD value of each point was generated from the mean SPAD of a total of five banana plants, i.e., the middle and four corner plants in each measurement point. Specifically, for a given measurement point, three leaves from the top of the canopies were selected from each of the five plants, and 20 SPAD measurements were conducted per leaf, with 60 measurements in total per plant. All measurements of the five plants were averaged to obtain the SPAD value for the corresponding ground measurement point. We performed the verification of the SPAD meter by quantifying the relationship between LCC determined in the laboratory and the SPAD value, but because of the limited experiment condition and operators, the LCC sampled from only 18 points were measured by chemical analysis (**Figure 2**). Several 1-cm circles were cut from each leaf sample. After weighing the fresh leaf weight, the samples were ground in 10 mL of 95% ethanol extract solution. After storing the solution in darkness for more than 24 h, the absorbance was measured with a UV-VIS spectrophotometer (Perkin-Elmer, Lambda 5, Waltham, MA, USA) at 649- and 665-nm wavelengths. The LCC were calculated using equations in Lichtenthaler (1987). From **Figure 2**, the SPAD value exhibited a strong exponential function relationship with LCC measured in the laboratory, which was in accordance with many published studies (Uddling et al., 2007; Parry et al., 2014), further indicating that it was reliable to use the SPAD meter for banana LCC measurement.

### 2.3 Extraction and screening of image features

#### 2.3.1 Extraction of vegetation index and textural feature

A series of image features including VIs and TFs were extracted from the hyperspectral images. A total of 20 VIs that were previously used for LCC estimation in the published literatures were selected in this study (**Table 1**). They were classified into original vegetation index (VI_org_) and red-edge vegetation index (VI_re_). Previously developed VI_re_ that has analogous form with corresponding VI_org_ was chosen to compare the performance for LCC estimation. Before the extraction, the hyperspectral images were firstly separated into banana plants and soil background by the statistic-based segment method. The process of this method was as follows: The NDVI was selected as a standardized way to assess whether a pixel observed was vegetation or not (Devadas et al., 2009). In general, NDVI ranging from 0.3 to 1.0 was considered as vegetation (Zhang et al., 2019). To find out the banana plants and soil in the two plots more accurately, the pixels only containing banana plants and the pixels only containing soil were selected and counted from the corresponding hyperspectral images, respectively. The result showed that the optimal thresholds of NDVI for separating banana plants and soil was set as 0.48 for plot 1 and 0.5 for plot 2, which were used as the mask files to extract the plants. The pixels with NDVI larger than 0.48 in plot 1 and larger than 0.5 in plot 2 were regarded as banana plants; the rest of the pixels were regarded as soil. It should be noted that the pixels only containing banana plants were used for the subsequent calculation of VIs and TFs. For the ground measurement points, the VIs and TFs were extracted as follows: 50*50 pixels region of interest (ROI) centered around the measurement point was manually selected, and then the mean value of VI or TF within the ROI was deemed to represent the corresponding measurement point.

**Table 1:** Vegetation indices used in this study.

Vegetation index abbreviation	Vegetation index	Formula		Reference	Bands used
Original vegetation index (VI_org_)					
SR	Simple ratio	$R_{nir}/R_{red}$		(Jordan, 1969)	802 nm, 682 nm
NDVI	Normalized difference vegetation index	$\left(R_{nir}-R_{red}\right)/\left(R_{nir}+R_{red}\right)$		(Sims and Gamon, 2002)	802 nm, 682 nm
GDVI	Green difference vegetation index	$R_{green}-R_{red}$		(Sripada, 2005)	550 nm, 682 nm
RDVI	Red difference vegetation index	$R_{nir}-R_{red}$		(Huete et al., 1994)	802 nm, 682 nm
MDVI	Modified difference vegetation index	$\left(R_{nir}-R_{red}\right)/\sqrt{R_{nir}+R_{red}}$		(Roujean and Breon, 1995)	802 nm, 682 nm
MSR	Modified SR	$\left(R_{nir}/R_{red}-1\right)/\sqrt{R_{nir}/R_{red}+1}$		(Chen, 1996)	802 nm, 682 nm
mNDVI	Modified NDVI	$\left(R_{nir}-R_{red}\right)/\left(R_{nir}+R_{red}-2R_{blue}\right)$		(Sims and Gamon, 2002)	802 nm, 682 nm, 446 nm
CI_green_	Green chlorophyll index	$R_{nir}/R_{green}-1$		(Gitelson et al., 2006)	770 nm, 510 nm
OSAVI	Optimized soil-adjusted vegetation index	$\left(1+0.6)(R_{nir}-R_{red})(R_{nir}+R_{red}+0.16\right)$		(Rondeaux et al., 1996)	802 nm, 682 nm
OSAVI_green_	Optimized soil-adjusted vegetation index with green	$\left(1+0.6)(R_{nir}-R_{green})(R_{nir}+R_{green}+0.16\right)$		(Qiao et al., 2022)	802 nm, 550 nm
SIPI	Structure insensitive pigment index	$\left(R_{nir}-R_{blue}\right)/\left(R_{nir}-R_{red}\right)$		(Penuelas et al., 1995)	802 nm, 446 nm, 682 nm
Corresponding red-edge vegetation index (VI_re_)					
SR_re_	Red-edge simple ratio	$R_{nir}/R_{red-edge}$	(Sims and Gamon, 2002)		802 nm, 706 nm
NDVI_re_	Red-edge normalized difference vegetation index	$\left(R_{nir}-R_{red-edge}\right)/\left(R_{nir}+R_{red-edge}\right)$	(Sims and Gamon, 2002)		802 nm, 706 nm
REDVI	Red-edge difference vegetation index	$R_{red-edge}-R_{red}$	(Sun et al., 2010)		710 nm, 682 nm
MDVI_re_	Modified difference vegetation index with red-edge	$\left(R_{nir}-R_{red-edge}\right)/\sqrt{R_{nir}+R_{red-edge}}$	(Qiao et al., 2022)		802 nm, 710 nm
MSR_re_	Modified red-edge SR	$\left(R_{nir}/R_{red-edge}-1\right)/\sqrt{R_{nir}/R_{red-edge}+1}$	(Wu et al., 2008)		802 nm, 710 nm
mNDVI_re_	Modified red-edge NDVI	$\left(R_{nir}-R_{red-edge}\right)/\left(R_{nir}+R_{red-edge}-2R_{blue}\right)$	(Sims and Gamon, 2002)		802 nm, 710 nm, 446 nm
CI_re_	Red-edge chlorophyll index	$R_{nir}/R_{red-edge}-1$	(Gitelson et al., 2006)		770 nm, 710 nm
OSAVI_red-edge_	Optimized soil-adjusted vegetation index with red-edge	$\left(1+0.6)(R_{nir}-R_{red-edge})(R_{nir}+R_{red-edge}+0.16\right)$	(Wu et al., 2008)		802 nm, 706 nm
MTCI	MERIS terrestrial chlorophyll index	$\left(R_{nir}-R_{red-edge}\right)/\left(R_{red-edge}-R_{red}\right)$	(Dash and Curran, 2004)		802 nm, 710 nm, 682 nm

For the TFs extraction, the hyperspectral images were first transformed by principal component analysis (PCA) with the aim of reducing the dimensionality, redundancy, and collinearity of data. In this study, we analyzed the TFs generated from the first three principal component (PC) images, i.e., the first PC image (PC1), the second PC image (PC2), and the third PC image (PC3), which contained more than 96% of the cumulative variance, hereinafter referred to as TFs-PC1, TFs-PC2, and TFs-PC3, respectively. Eight TFs were extracted to evaluate their correlation with LCC (**Table 2**). They were based on the gray-level co-occurrence matrix defined by Haralick et al. (1973). A 3 × 3 calculation window was chosen when calculating the TFs, which could capture more local details and facilitate to detect subtle changes of image texture (Yue et al., 2019).

#### 2.3.2 Screening of image features

Pearson correlation coefficient (*r*) and maximum information coefficient (MIC) were used to evaluate the correlation between VIs or TFs and LCC. Pearson correlation coefficient is a widely used index which can measure the linear correlation between two variables, with the *r* value ranging from -1 to 1, while MIC was explored to evaluate the linear and nonlinear between variables; the range is 0 to 1. The higher the absolute value of *r* (|*r*|) or MIC, the better correlation of the VI or TF with respect to LCC. In this study, the VI or TF who has |*r*| or MIC higher than 0.8 was selected as one of the potential predictors for banana LCC using MLRAs method.

### 2.4 LCC estimation modeling and validation

In this section, we propose two types of methods for assessing banana LCC, i.e., two-pair image feature combination method and multivariable image feature combination using MLRAs. Model validation was also included.

#### 2.4.1 LCC estimation based on two-pair image feature combination

The SR, NDVI, and DVI are three types of earlier proposed and the most classic and the most widely used formulas in leaf biochemical and physiological parameters estimation, which are composed of two spectral reflectance or features. Inspired by this, we calculated all possible VI and TF combinations in types of SR, NDVI, and DVI (referred to as two-pair image feature combination) for banana LCC assessment. Linear regression was adopted to model the relationship between LCC and each image feature combination, and their performances were tested to determine the best two-pair image feature combination for LCC estimation. They were defined as Equations 1–3). We consequently obtained a total of 160 image feature combinations for each type. All of the calculations were implemented using MATLAB R2021b (The MathWorks, Inc., Natick, MA, USA).


(1) $$SR-type=\frac{TF}{VI}$$

(2) $$NDVI-type=\frac{VI-TF}{VI+TF}$$

(3) DVI−type=VI−TF

#### 2.4.2 LCC estimation using multivariable image feature combination based on MLRAs

Four MLRAs, including PLSR, ARS, SVR, and GPR, were employed to combine more than two image features for LCC estimation. The VIs, original Vis, and TFs combinations, as well as the selected VIs and TFs combinations optimized by Pearson correlation and MIC, were respectively used as input variables in these MLRAs. The ARS, SVR, and GPR models were conducted to build nonlinear relationships between input variable and LCC, while the PLSR model was carried out to build their linear relationship.

PLSR is a bilinear calibration method for retrieving vegetation biochemical parameters. It integrates multiple linear regression, least square regression, and principal component analysis, which compresses the independent variables (e.g., multiply image features) into several latent variables with the strongest explanation to the model system and finally reduces the multi-collinearity problem of input variables and influence of data noise on the regression model (Hansen and Schjoerring, 2003).

ASR is a basic function-based nonparametric regression, which was firstly developed by Friedman and Roosen (1995). ASR has the ability of automatically identifying the most relevant variables available from remote sensing data and of handling variable interactions, which is crucial for integrating multi-type data (e.g., VIs and TFs). It also provides an explicit expression for prediction model, making it easier to interpret the relationships between input features and vegetation parameters.

SVR was derived from the statistical learning theory proposed by Cortes and Vapnek (1995). It can effectively capture the complex nonlinear relationships between remote sensing data and vegetation parameters by employing kernel functions to map input variables into a high-dimensional feature space. Additionally, SVR relies primarily on support vectors for training, making it useful when the number of samples was limited in biochemical parameters estimation (Guo et al., 2022).

GPR is a probabilistic approximation to nonparametric kernel-based regression. It has been proven to have significant advantages in vegetation parameter retrieval due to its powerful nonlinear modeling capability, small sample adaptability, and ability to quantify prediction uncertainty (Campos-Taberner et al., 2016; Upreti et al., 2019). It offers an explicit form of the predictive model, which establishes a nonlinear relation between the input (e.g., VIs and TFs) and the output variable (i.e., LCC). Moreover, GPR is particularly suitable for scenarios with limited ground-measured data and supports the integration of multi-source and high-dimensional input features.

#### 2.4.3 Model validation

The validity of LCC estimation models was assessed based on a k-fold (*k* = 10) cross-validation procedure, which splits the dataset into 10 equal-sized subsets. In each iteration, nine subsets were used as the training set, and the remaining one was used as the validation set to evaluate the models. The coefficient of determination (*R* ^2^) and the root mean square error (RMSE) of the measured and predicted LCC values were used to evaluate the prediction ability of the models based on two-pair image feature combination method and multivariable image feature combination using MLRAs.

## 3 Results

### 3.1 Correlation analysis between image feature and LCC

**Figure 3:**
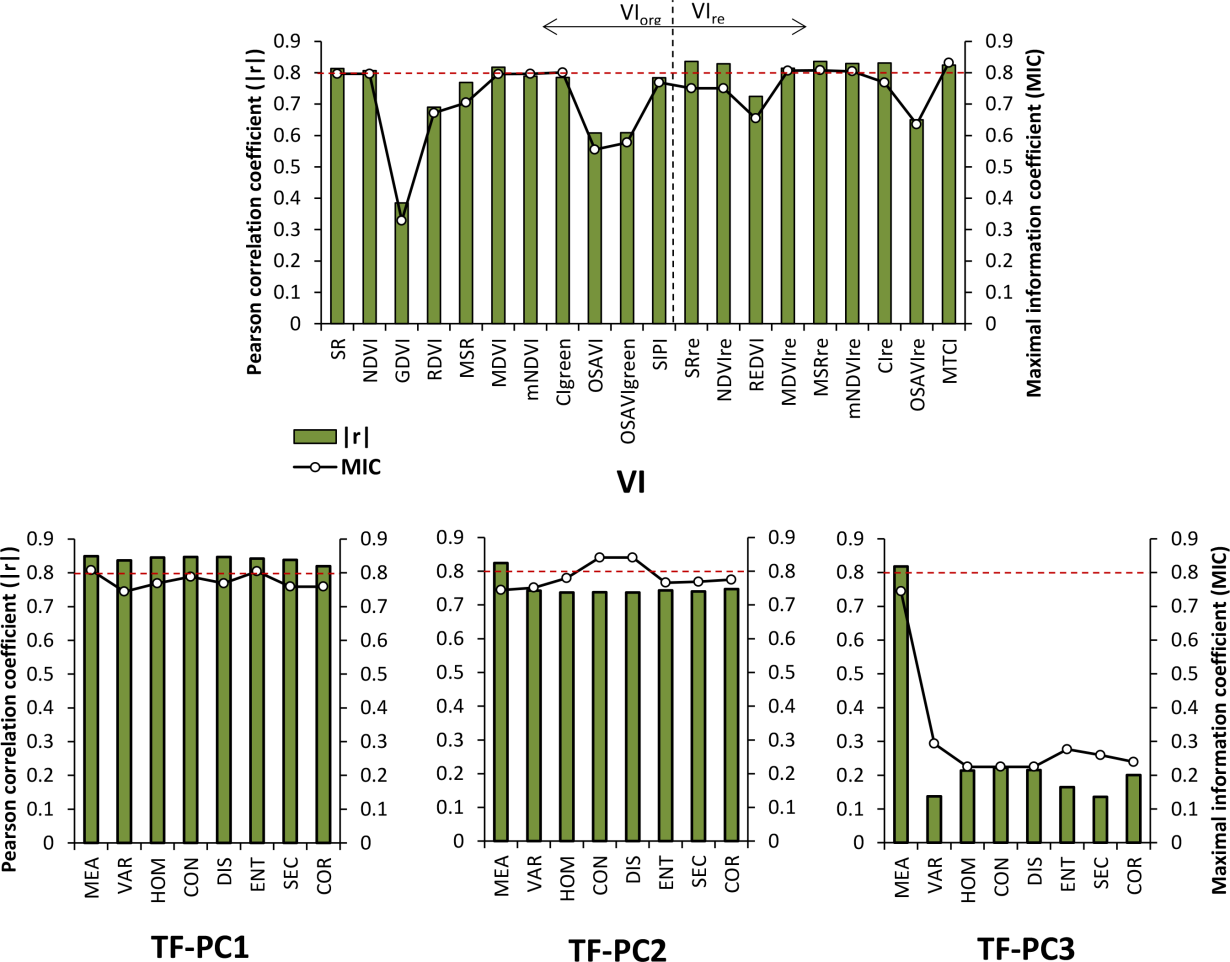
Pearson correlation coefficient (|*r*|) and maximal information coefficient (MIC) between different types of VIs and TFs vs. LCC. The green histogram indicates the value of |*r*|, the black broken line indicates the value of MIC, and the red dashed line indicates |*r*| = 0.8 or MIC = 0.8.

The correlations between LCC and all VIs in **Table 1** and all TFs extracted from three PC images in **Table 2** were calculated and analyzed. **Figure 3** shows the |*r*| and MIC values for different types of VIs and TFs. The values of |*r*| or MIC larger than 0.8 were selected and presented in **Table 3**. From the correlations of different VIs with respect to LCC, as expected, a majority of VI_re_ were superior to the corresponding VI_org_ with similar forms. For instance, the GDVI showed weakness in correlating LCC with |*r*| of 0.38 and MIC of 0.33, while the REDVI dramatically increased by 88% and 98% respectively, compared to the GDVI. The values of MIC of the MDVI_re_ and OSAVI_re_ were also improved greater than 14% in comparison of the MDVI and OSAVI. This result highlighted a strong potential for use in banana LCC estimation with red-edge spectral band. The best VI was MSR_re_, which generated the correlation of 0.836 with LCC. For the TFs, the TFs-PC1 were generally correlated better with LCC than the TFs-PC2 and TFs-PC3, owing to the larger values of both |*r*| and MIC. From **Table 3**, we found that all eight TFs obtained in PC1 image were selected using Pearson correlation, while none of the TF extracted in PC3 image reached the threshold of MIC. The MEA was selected not only in all PC images by Pearson correlation method but also in PC1 image by MIC.

**Table 2:** Texture features used in this study.

Texture feature abbreviation	Texture feature	Formula
MEA	Mean	$MEA=\sum_{i,j=1}^{G}ip(i,j)$
VAR	Variance	$VAR=\sum_{i=1}^{G}\sum_{j=1}^{G}{\left(i-u\right)}^{2}p(i,j)$
HOM	Homogeneity	$HOM=\sum_{i=1}^{G}\sum_{j=1}^{G}\frac{p(i,j)}{1+{\left(i-j\right)}^{2}}$
CON	Contrast	$CON=\sum_{i=1}^{G}\sum_{j=1}^{G}{\left(i-j\right)}^{2}p(i,j)$
DIS	Dissimilarity	$DIS=\sum_{i=1}^{G}\sum_{j=1}^{G}p(i,j)|i-j|$
ENT	Entropy	$ENT=-\sum_{i=1}^{G}\sum_{j=1}^{G}p(i,j)\log p(i,j)$
SEC	Second moment	$SEC=\sum_{i=1}^{G}\sum_{j=1}^{G}p{\left(i,j\right)}^{2}$
COR	Correlation	$COR=\sum_{i=1}^{G}\sum_{j=1}^{G}\frac{\left(i-j)(j-i)p(i,j\right)}{\sqrt{VAR_{i}\times VAR_{j}}}$

In the formulas, *i* and *j* represent the row number and column number of the image, respectively, and *p*(*i,j*) represents the relative frequency of two neighboring pixels.

**Table 3:** Selected VIs and TFs using Pearson correlation coefficient (|*r*|) and maximal information coefficient (MIC).

Image feature	Selected by |*r*| value	Selected by MIC value
VI	SR, NDVI, MSR, SR_re_, NDVI_re_, MDVI_re_, MSR_re_, mNDVI_re_, CI_re_, MTCI	SR, NDVI, MSR, mNDVI, CI_green_, MDVI_re_, MSR_re_, mNDVI_re_, MTCI
TF-PC1	MEA, VAR, HOM, CON, DIS, ENT, SEC, COR	MEA, ENT
TF-PC2	MEA	CON, DIS
TF-PC3	MEA	–

### 3.2 Banana LCC estimation using two-pair feature combination of VI and TF

**Figure 4:**
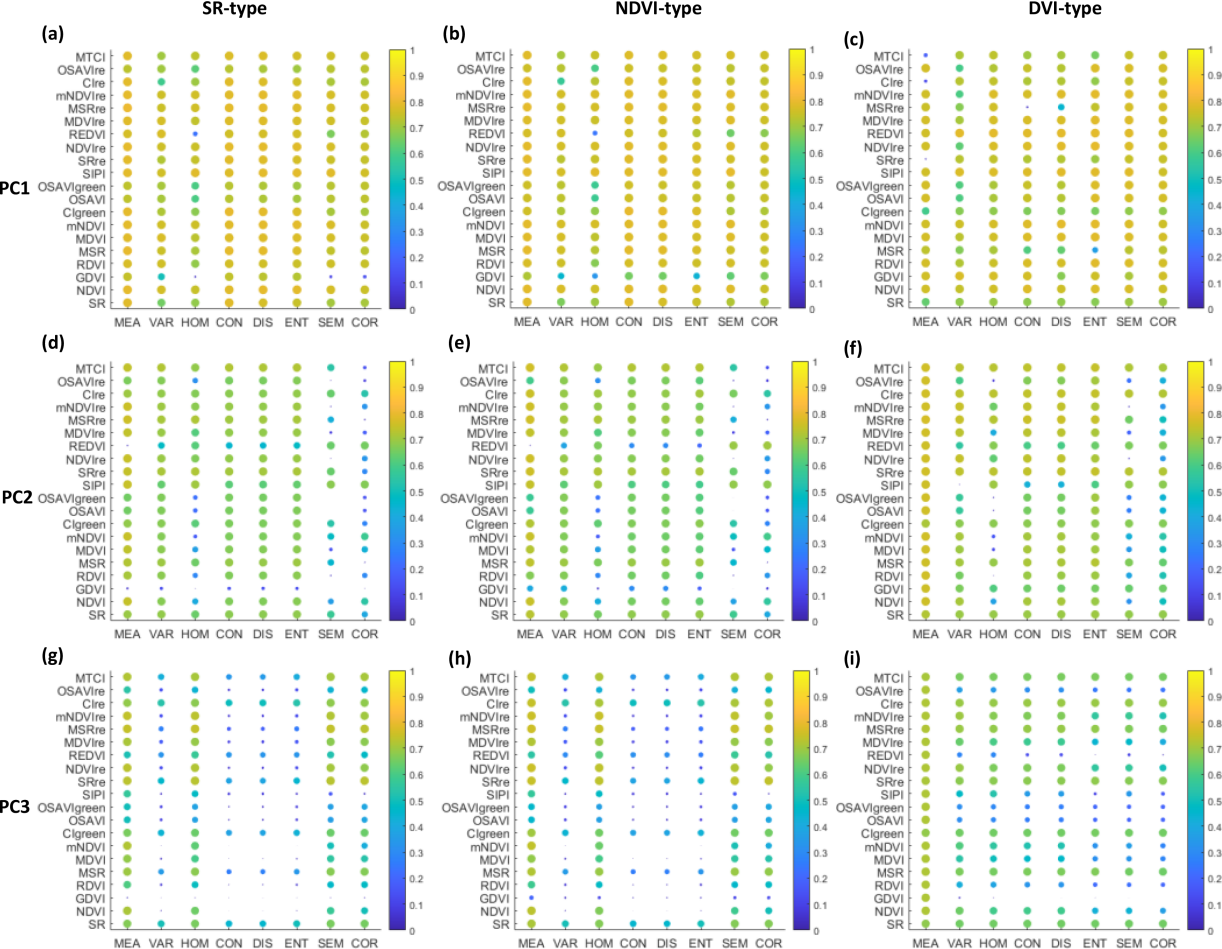
Coefficient of determination (*R* ^2^) based on the linear relationship between LCC and all possible two-pair feature combinations of VI and TF extracted from PC1 **(A–C)**, PC2 **(D–F)**, and PC3 **(G–I)** images in types of SR, NDVI, and DVI.

**Figure 4** shows all coefficient of determination (*R* ^2^) values based on the linear regression analyses of LCC against all possible two-pair combinations of the VI and TF used in types of SR, NDVI, and DVI. The closer to yellow and the higher the size of the scatter, the larger *R* ^2^ value and better accuracy of the model derived from the image feature combination. The results indicated that for all the three types, image feature combinations calculated from the TFs-PC1 paired with VIs exhibited the strongest relationship with LCC (**Figures 4A–C**) and then those calculated from the TFs-PC2 (**Figures 4D–F**) followed by the TFs-PC3 (**Figures 4G–I**). This means that textural information on the PC1 image contributed more in LCC determination compared to that on the PC2 and PC3 images. Furthermore, it should be noteworthy that in comparison to other TFs, the MEA combined with almost all VIs preserved higher sensitivity to LCC variability, which was coincident with the results in **Figure 3** where the MEA is showing better correlation and also being selected from the PC1 and PC2 as well as PC3 images. This phenomenon was more obvious when extracting the MEA from PC2 and PC3 images, with the size and color of scatters revealing relatively high *R* ^2^ values (**Figures 4D–I**).

From **Figures 4A–C**, almost all image feature combinations showed similar patterns among the SR, NDVI, and DVI types. A high degree of image feature combinations constructed by the TFs-PC1 and VIs behaved well against LCC. The MSR_re_ together with MEA were thereinto selected from every 160 feature combinations in each type due to their outstanding performances in capturing variations in CCC with higher *R* ^2^ than 0.75. The result is shown in **Table 4**. It can be seen that the most effective feature combination was provided by the ratio of MEA to MSR_re_ (referred to as MEA/MSR_re_), which explained 78.9% of the variation in LCC, implying that the ratio clearly combines the abilities of the given VI and TF responding to LCC. Whereas feature combinations that showed weakness in characterizing LCC were different among the tree types, the *R* ^2^ values obtained using the HOM/GDVI, SEM/GDVI, MEA−SR_re_, and CON−MSR_re_ were less than 0.1.

**Table 4:** Optimal two-pair feature combination selected in types of SR, NDVI, and DVI, linear relationship, and coefficient of determination (*R* ^2^) between optimal feature combination and LCC.

Type	PC image	Optimal two-pair feature combination	Model	*R* ^2^
SR-type	PC1	$MEA/MSR_{re}$	y=4.4405x+52.45	0.789
NDVI-type	PC1	$\left(MEA-MSR_{re}\right)/\left(MEA+MSR_{re}\right)$	y=−1.7169x+76.08	0.77
DVI-type	PC1	$MEA-MSR_{re}$	y=−197.89x+243.61	0.756

*x* and *y* in the “Model” column refer to the optimal two-pair feature combination and LCC, respectively.

### 3.3 Banana LCC estimation using multivariable image feature combinations

PLSR, ARS, SVR, and GPR methods were employed to combine more than two image features for LCC estimation. Three variable groups were included, i.e., VIs, original Vis, and TFs combinations, and selected VIs and TFs combinations from Pearson correlation as well as from MIC as shown in **Table 3**. To assess the predictive capabilities of the models, *R* ^2^ and RMSE were calculated for all of the modeling results (**Table 5**). Overall, the models using all VIs showed a relatively moderate performance, with *R*² values around 0.615 to 0.63 and RMSE values around 2.65 to 2.75. In comparison with all VIs, the models based on VI_re_ provided a more accurate estimation in terms of *R* ^2^ and RMSE values when using the four MLRAs, with *R*² increased to a range of 0.63 to 0.67 and RMSE decreased to a range of 2.492 to 2.643. More importantly, the image feature combinations, which not only included spectral information but also image textures, dramatically improved the estimation results for LCC across the three input groups compared to using the VIs alone. However, MLRAs showed varying estimation performances for different feature combinations.

**Table 5:** Predicted LCC results using PLSR, ARS, SVR, and GPR with different input variables.

Variable group	Input variable	Number of features	PLSR		ARS		SVR		GPR	
			*R* ^2^	RMSE	*R* ^2^	RMSE	*R* ^2^	RMSE	*R* ^2^	RMSE
Original features	All VIs	20	0.615	2.696	0.63	2.652	0.63	2.652	0.6	2.754
	All VI_re_	9	0.63	2.643	0.67	2.492	0.65	2.542	0.66	2.538
	All VIs+TFs-PC1	28	**0.756**	2.082	**0.75**	2.064	**0.75**	2.169	**0.76**	2.09
	All VIs+TFs-PC2	28	0.725	2.262	0.735	2.326	0.698	2.369	0.71	2.307
	All VIs+TFs-PC3	28	0.726	2.261	0.71	2.34	0.71	2.248	0.669	2.499
Features selected by Pearson correlation (|*r*| > 0.8)	Selected VIs+TFs-PC1	18	**0.753**	2.041	**0.767**	2.078	**0.765**	2.09	**0.768**	2.074
	Selected VIs+TFs-PC2	11	0.718	2.176	0.704	2.345	0.737	2.209	0.735	2.218
	Selected VIs+TFs-PC3	11	0.735	2.22	0.736	2.214	0.732	2.231	0.725	2.258
Features selected by MIC (MIC > 0.8)	Selected VIs+TFs-PC1	11	0.749	2.162	0.748	2.168	**0.767**	2.124	**0.776**	2.04
	Selected VIs+TFs-PC2	11	0.711	2.318	0.699	2.368	0.729	2.245	0.672	2.467

*R* ^2^ values higher than 0.75 are in bold. All VIs+TFs-PC1, image feature combination combined by all VIs and all TFs-PC1; Selected VIs+TFs-PC1, selected feature combination combined by selected VIs and selected TFs-PC1 from Pearson correlation coefficient (|*r*| > 0.8) or maximal information coefficient (MIC > 0.8); TFs-PC1, textural features extracted from the first principal component images; TFs-PC2, textural features extracted from the second principal component images; TFs-PC3, textural features extracted from the third principal component images.

From the results of original feature combination and two selected feature combination groups, it could be observed that the examination of different feature combinations input showed that the combinations of TFs-PC1 and VIs resulted in better LCC estimates than the combinations of TFs-PC2 and VIs, with almost all *R* ^2^ values exceeding 0.75. Similar performances with models using TFs-PC2 can be found in the models using TFs-PC3. With respect to the MLRAs used, the GPR method acquired a slightly better accuracy when considering the VIs+TFs-PC1 as input variable in the three feature combination groups.

As expected, the VIs+TFs-PC1 combination selected using Pearson correlation method provided a robust improvement, especially for the ARS, SVR, and GPR models, with *R*² ranging from 0.765 to 0.768 and RMSEs close to 2.074. Meanwhile, the desirable result was also seen for models derived from the VIs+TFs-PC1 combination selected through MIC, especially the GPR model, which outperformed all other MLRAs, demonstrating that it was the best model for LCC estimation.

### 3.4 Evaluation of LCC prediction using UAV hyperspectral images

**Figure 5:**
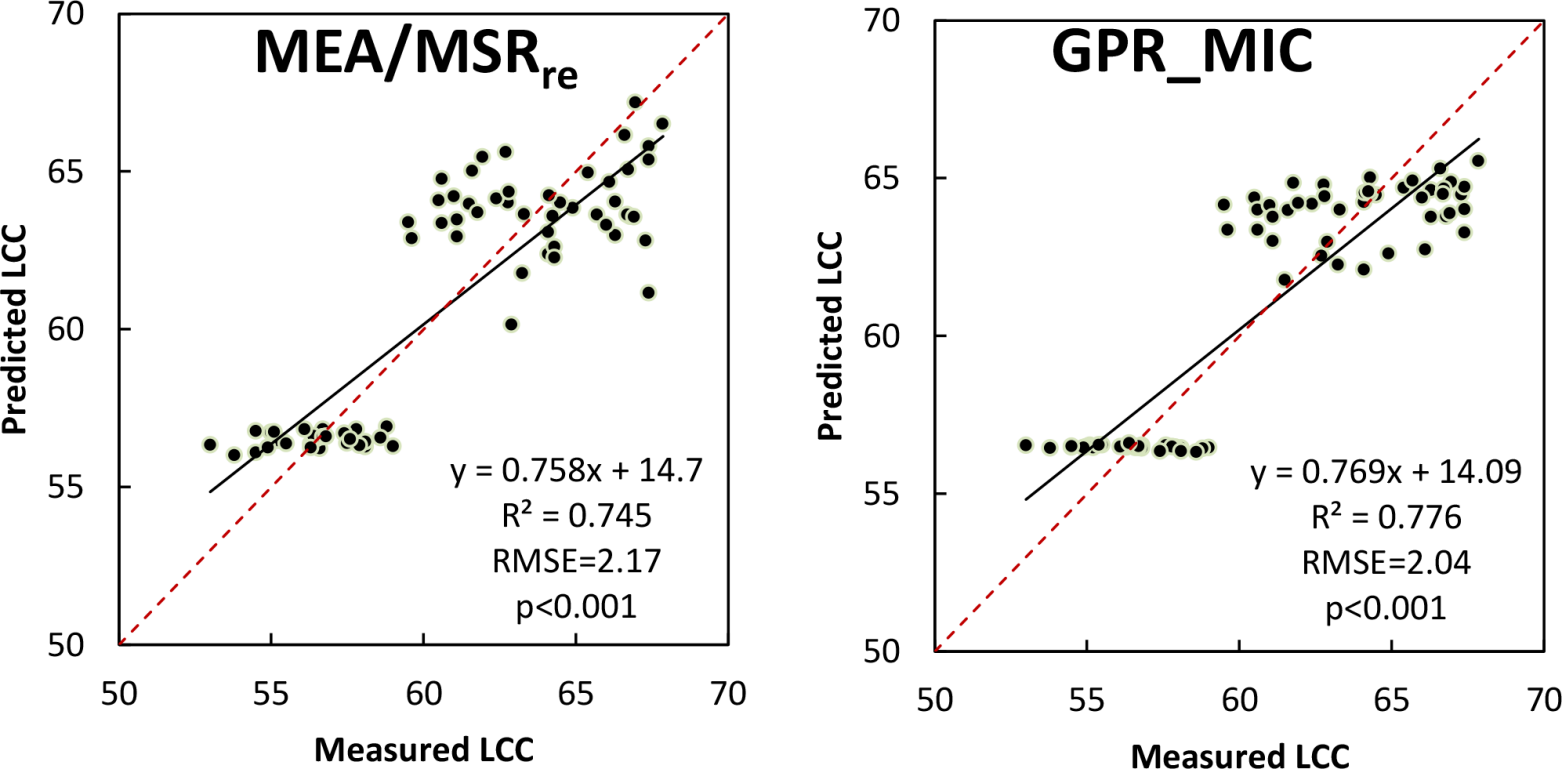
Comparison between predicted LCC from UAV hyperspectral reflectance data and ground-measured LCC using the best two-pair MEA/MSR_re_ combination (left) and GPR model derived from selected VIs+TFs-PC1 extracted through the MIC method (right).

We compared the performance of the best two-pair image feature combination and that obtained in a multivariable calibration based on GPR algorithm for LCC prediction, i.e., models derived from the MEA/MSR_re_ and the selected VIs+TFs-PC1 extracted through the MIC method. **Figure 5** shows the scattering plot between LCC estimated from UAV hyperspectral reflectance data and LCC measured in the field campaign. We found that the predictive capability of the two models seems satisfactory, which had led to high coefficients of determination (*R* ^2^ > 0.74) and good RMSE values, and they all reached the 0.001 significance level. However, judging by the scattering point distribution, a slight dispersion of the MEA/MSR_re_ model occurred; prediction using the GPR model achieved the best result, with the highest *R* ^2^ of 0.776 and the lowest RMSE of 2.04.

**Figure 6:**
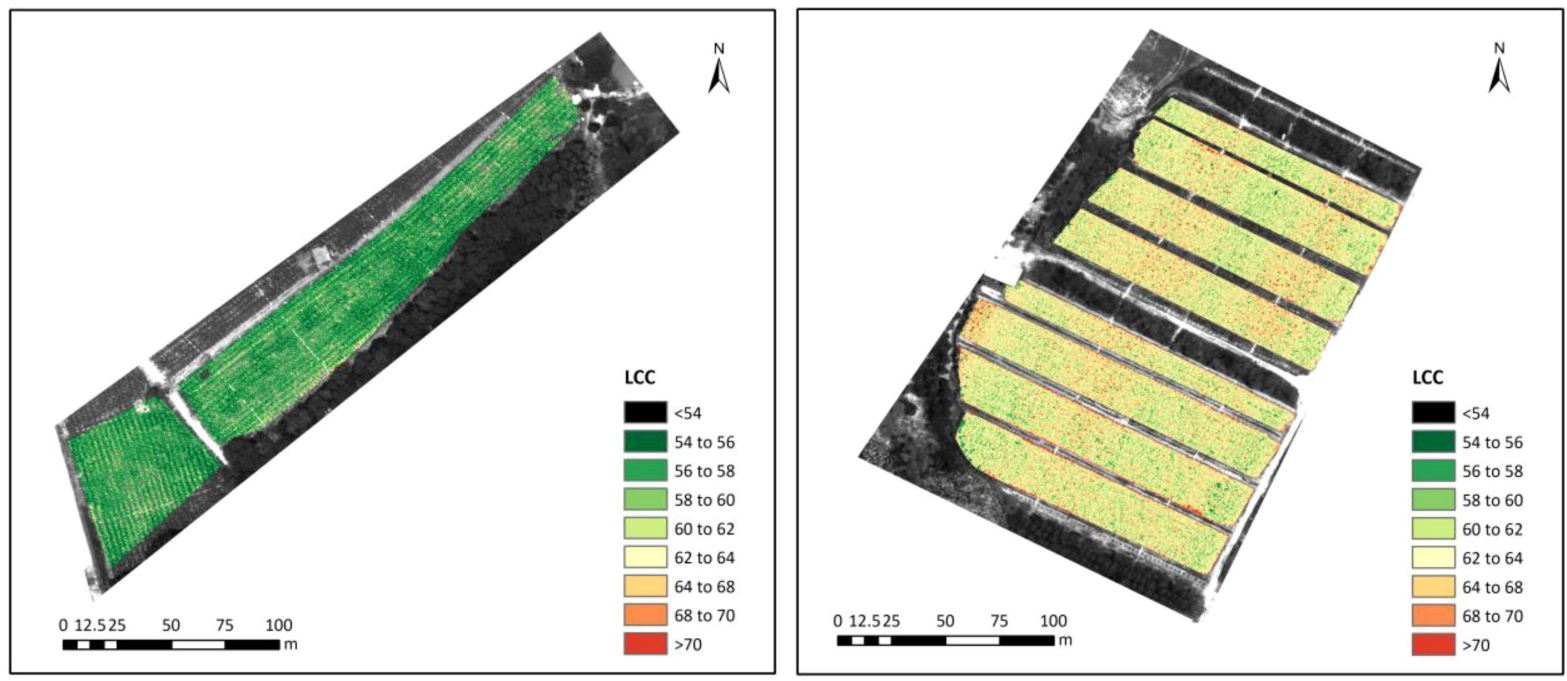
Spatial distributions of banana LCC in plot 1 (left) and plot 2 (right) mapped using the best GPR model.

Based on the results presented above, the GPR model was applied to UAV hyperspectral images to map chlorophyll status over the two large plots of banana, as shown in **Figure 6**. Because they corresponded to the LCC distribution at different growth stages of banana, various spatial variability in each plot was exhibited. A first observation across the different sites was that the banana LCC in plot 1 was overall homogeneous at the leaf development stage (**Figure 6**, left). Nevertheless, at the fruit development stage, a more obvious spatial heterogeneity of LCC appeared in plot 2 (**Figure 6**, right) due, in large part, to the different degrees of nutritional absorption and migration at the later stage of the banana. Moreover, the dynamic changes of LCC were revealed; they gradually increased as the progress of growth. The range of LCC at leaf development stage was concentrated from 54 to 60, while the LCC at fruit development stage dominated the range of 58 to 70.

## 4 Discussion

The assessment and monitoring of banana LCC status and spatial distribution are of importance for addressing crucial issues, such as growth monitoring, nutritional stress, and management practices. In this study, we estimated LCC by various feature combinations of spectral and textural information derived from UAV hyperspectral images for banana at different growth stages. The VI method is widely used in quantifying vegetation parameters. However, many literatures have indicated that soil background has a strong influence on canopy reflectance and the derived VIs relating to leaf parameters, especially at the early growth stages (Zha et al., 2020). A meaningful process we conducted to the original UAV hyperspectral images was to remove the soil background pixels by statistic threshold segment method. Then, a series of VIs and TFs were extracted based on pixels only containing vegetation, further ensuring the reliability of LCC estimation. However, relative experiments should be conducted to further verify the specific impact of soil background on banana LCC quantification in the future.

**Figure 7:**
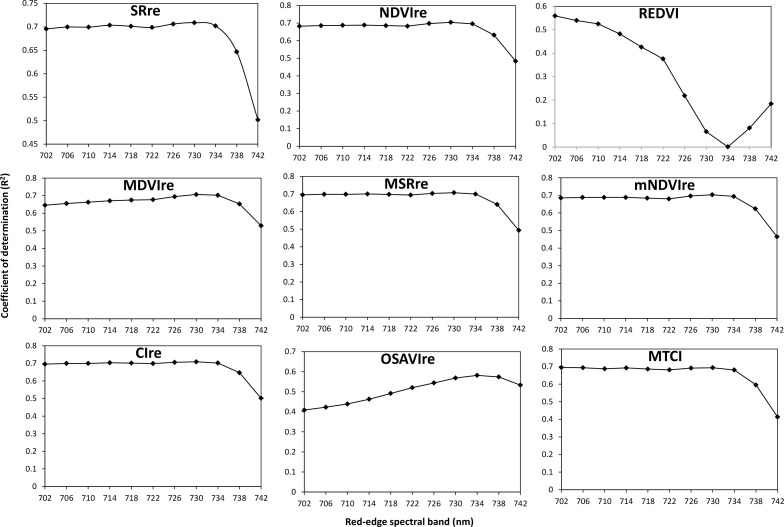
*R* ^2^ values of linear relationships between LCC and the VI_re_ derived from the red-edge spectral band from 702 to 742 nm.

The red-edge spectral bands located between 700 and 740 nm with being not static but rather shifting during vegetation stress or in a good condition was proven to have the potential for improving LCC estimation for a variety of vegetation, such as maize, wheat, maple, sugar beet, etc (Gitelson et al., 2006; Wu et al., 2008; Jay et al., 2017; Kong et al., 2022), which inspired the inclusion of red-edge bands into not only airborne but also satellite sensors. The VI_re_ used in our study was likewise expected to perform better for banana. It is interesting to note that a desirable result was obtained when using UAV hyperspectral data. It is pronounced in banana LCC determination where the VI_re_ showed higher sensitivity as compared with the original VI with a similar form, which often suffers from saturation problems (Wu et al., 2008). To identify the specific band in the red-edge region from UAV hyperspectral image that can substantially make the VI_re_ achieve the best result for banana, we applied the spectral reflectance that ranged from 702 to 742 nm to replace the original red-edge band contained in the VI_re_ one by one and further compared their performances in LCC estimation. The *R* ^2^ values of linear relationships with LCC are presented in **Figure 7**. An important information revealed in the figure was that the trends of all VI_re_ curves, except for the REDVI and OSAVI_re_, were generally similar, showing the best correlation with LCC when 730-nm red-edge band participated in the regression model. This implied that the red-edge band centered mostly at 730 nm could help in the design of an index or a sensor band that could better estimate the LCC for banana.

In addition to individual VI, the linear two-pair feature combination and nonlinear multivariable MLRAs were independently applied to combine the VI and TF features derived from hyperspectral images for LCC assessment. Based on **Figure 4** and **Table 5**, the more accurate estimation results were generated when the TFs (especially the MEA) were involved in LCC models compared to models only using spectral information. Guo et al. (2022); Yue et al. (2019), and Reddersen et al. (2014) also reported that combing VIs and TFs can lead to better LCC and aboveground biomass estimates for crops and grassland. Our outcomes open the possibility to couple the potential of spectral and textural features for further improving the accuracy of LCC retrieval for tropical fruit tree (i.e., banana) at the field scale. In the present study, eight TFs were selected, and a total of 24 TFs were extracted from the first three images after principal component analysis. However, different performances of VI paired with TF extracted from different PC images were obtained; the combined uses of VI and TF-PC1 achieved better results than others combined by TF-PC2 and TF-PC3 whether in linear relationships or nonlinear MLRAs. One of the main reasons for such results is that the growing of banana plants could cause the changes in TFs of UAV image data. The TFs extracted from PC1 image captured more information related to the spatial resolution of the dark and bright areas of the image (Haralick et al., 1973), facilitating the accuracy of LCC prediction.

Compared to the two-pair image feature combination method, MLRAs using a combination of multiple VIs and TFs-PC1 as input had a slightly better ability for LCC prediction, especially the VIs+TFs-PC1 selected by Pearson correlation and MIC. This was expected since they utilized more features and nonlinear transforms. Among all MLRAs, the GPR model based on not only the original but also the selected feature combinations stood out as being the most accurate than the rest of the MLRAs (**Table 5**), suggesting that it is the optimum algorithm for banana LCC. This is in accordance with previous studies which reported that the GPR satisfied in varied leaf parameter retrieval using airborne or spaceborne satellite data (Verrelst et al., 2013a; Upreti et al., 2019; Zhou et al., 2020). The GPR with selected VIs+TFs-PC1 by MIC as input provided the best result in terms of both high accuracy and low error (**Figure 5**, right) for LCC ground validation test. In essence, GPR is based on non-parametric regression in a Bayesian framework. It builds models by taking fully into account the characteristics of the data itself, making it affordable to deal with complex, nonlinear, or irregular data (Campos-Taberner et al., 2016). Furthermore, the model was earlier evaluated as a potential predictor with a relatively small dataset (Verrelst et al., 2013a), which was another reason that ensuring it still had an outstanding performance when the number of banana ground-based samples was limited in our study. More importantly, along with pixelwise estimation maps for banana LCC at two growth stages, GPR can provide the accompanying confidence intervals, which was a significant advantage over other competitive MLRAs (e.g., PLSR, ASR, and SVR) because these confidences put forward some insight in the robustness and reliability of the LCC retrieval (Verrelst et al., 2013b). Furthermore, a total of 11 image features were screened by the MIC, including five original vegetation indices, four red-edge vegetation indices, and two texture features extracted from PC1 images. The selected VIs contained the narrow bands in red, red-edge, and near-infrared spectral regions which were proven to be sensitive and closely related to the LCC (Gitelson et al., 2006; Wu et al., 2008; Zhou et al., 2024). The introduced green band could suppress the saturation phenomenon of the relationship of absorptions *versus* LCC and is resistant to atmospheric effects (Gitelson et al., 1996, Gitelson et al., 2003). The selected two TFs, i.e., MEA and ENT, provided a more comprehensive analysis of texture features of UAV images, helping to understand the global brightness distribution and texture complexity (Haralick et al., 1973). This suggests that the inclusion of multiple effective image features in the regression model allowed for the incorporation of more valid spectral and textural information related to the LCC variable, resulting in improved estimation for banana. Even though the GPR model based on VIs+TFs-PC1 selected using MIC achieved a relatively high accuracy with limited samples of LCC, it is still needed to collect more ground-measured samples throughout the whole growth periods of banana in future research.

## 5 Conclusion

The analyses of this study indicated the potential of UAV hyperspectral images in efficient LCC monitoring for tropical fruit trees, i.e. banana plants, across different growth stages. We first analyzed if using the red-edge bands in UAV hyperspectral data improved the estimation of banana LCC over conventionally used original red or green bands in VIs. The result demonstrated that the VI_re_ presented better correlation and achieved higher sensitivity in LCC estimation based on MLRAs compared to the VI_org_, expanding the positive role of red-edge bands in assessing LCC for banana. In addition to VIs, several TFs, especially the MEA, also showed satisfactory correlations with LCC. To investigate the contribution of VIs integrated with TFs, on one hand, we proposed a comprehensive two-pair VI and TF combination method to explore the best two-pair feature combination for estimating LCC; on the other hand, MLRAs with multivariable groups containing VIs and TFs as input were also developed and applied. We found that the combination of VI and TF significantly improved the accuracy of LCC retrieval in comparison to using VI alone. The most robust two-pair feature combination was MEA/MSR{sb}{/sb}_re_, and the GPR model using the selected VIs+TFs-PC1 extracted through MIC as input variable outperformed the other MLRAs (i.e., PLSR, ASR, and SVR). They improved the prediction accuracy with *R* ^2^ of 0.745 (*p* < 0.001) and 0.776 (*p* < 0.001), respectively, also implying that the latter model was the most suitable one for quantifying banana LCC. This study provides insights into the remote estimation of LCC for tropical fruit trees. Our proposed retrieval approaches by combing the spectral and image textural features could offer great possibilities for more accurate diagnosing of nutritional status and providing practical guidance for precision fertilization.

## Data Availability

The original contributions presented in the study are included in the article/supplementary material. Further inquiries can be directed to the corresponding authors.
